# Supplementary material for: Outdoor recreational activity experiences improve psychological wellbeing of military veterans with post-traumatic stress disorder: Positive findings from a pilot study and a randomised controlled trial
Source: PLoS One. 2020 Nov 25;15(11):e0241763. doi: 10.1371/journal.pone.0241763 (PMC7688151; doi:10.1371/journal.pone.0241763)
Supplement: S1 File — (DOCX) [file pone.0241763.s001.docx]

**Title: Exploring the effect of outdoor recreational activity experiences on the psychological wellbeing of military veterans with post-traumatic stress disorder (PTSD): a pilot study.**

**Names of researchers**: Dr Nicholas Cooper, Dr Mark Wheeler, Prof. Sheina Orbell, Dr Leanne Andrews, Prof. Jamie Hacker Hughes, Dr Tim Rakow, Dr Marie Juanchich.

**Background, Aims & Objections**: Exposure to the natural environment is being increasingly seen as a method for increasing wellbeing and psychological health in both clinical and non-clinical populations. Recently, reports in the literature also suggest that outdoor pursuits involving opportunities for social interaction and bonding may be particularly beneficial to Armed Forces Veterans experiencing Post-Traumatic Stress Disorder (PTSD). In particular, organized, social fishing trips have been shown to reduce psychological measures of stress in US veterans (Vella et al., 2013). Consequently, the current study aims to assess the impact of an outdoor pursuit programme (contrasting social angling with other outdoor experiences – equine husbandry, archery and falconry) on the psychological wellbeing of UK Veterans. Wellbeing is assessed in terms of PTSD symptoms, depression, anxiety and stress.

**Hypotheses**: It is hypothesized that participants will experience a reduction in PTSD-related symptomology and an increase in subjective well-being as a consequence of a short, outdoor, recreational activity.

**Participants**: Inclusion criteria – military veterans with PTSD. Exclusion criteria – Not currently receiving psychological therapy for PTSD. Sample size – 30 (based on those meeting eligibility criteria from local sample).

**Recruitment**: Participants to be recruited from a population of service users registered at local military welfare services.

**Design**: The experiment employed a pretest - posttest within participant design (time: pre-intervention, 2 weeks post-intervention, 4 months post-intervention) with one between groups factor (type of activity: angling, equine, falconry and archery). Each activity intervention is designed to deliver an outdoor recreational activity in a peer group context and to facilitate opportunities to socialise and to discuss military experience or PTSD experience if the participant so wishes. The three different activity interventions will run sequentially and employ the same eligibility criteria, recruitment process and evaluation.

**Randomisation**: Participants will be randomly allocated to one of the three levels of the between groups factor (angling v equine husbandry v archery/falconry). They will be sequentially numbered and allocated to condition by means of an online blocked randomisation tool.

**Description of Procedure**: Dependent variables - The programme entails psychological profiling, in the form of the following questionnaire assessments: PCL-M (post-traumatic stress disorder measure for military personel), GAD-7 (anxiety measure), PHQ-9 (depression measure), PSS (perceived stress scale) at 2 weeks prior to intervention, 2 weeks and 4 months post intervention for time points for all three groups).

The equine husbandry intervention protocol – Day 1 am – arrival at stables. Training and practice of equine husbandry, riding instruction and social interaction. Break for lunch. Day 1 pm – further equine husbandry, riding instruction and social interaction. Riding of horses. De-brief and instructions on how to keep in contact via social media etc.

The archery/falconry intervention protocol – Day 1 am – arrival at venue. Acquaintance with the birds and equipment, hunting with the birds and social interaction. Break for lunch. Day 1 pm – Further falconry. Archery and social interaction. The archery will be conducted under the tuition of fully qualified and insured instructors from the falconry centre.

The angling intervention protocol - Day 1 am – arrival at fishing lake, set-up equipment, health and safety briefing. Day 1 pm – fishing instruction, fishing & social interaction, evening meal. Day 2 am – fishing & social interaction. Day 2 pm – fishing & pack up equipment, de-brief and instructions on how to keep in contact via social media etc.

General intervention details - The interventions are designed to deliver an outdoor recreational experience involving tuition in a peer group context. Attention is given to creating opportunities for participants to interact with each other. In each context, the venue will be made available exclusively to the veterans for the duration of the experience. Professional coaches (in angling, horse husbandry and riding, falconry and archery) provide instruction and are available at a ratio of two participants to one coach.

Aside from the different activities pursued, each outdoor activity experience contains the same common elements. Participants are transported to the venue by minibus. On arrival at the venue, a health and safety briefing will take place. Participants are then allocated to coaches and provided with equipment (and designated horse in the case of equine, and designated fishing spot around a lake in the case of angling). Participants collaborate in setting up a communal area for the purpose of socialising, eating and taking warm drink breaks. Food (e.g. sausages, burgers, chicken, salad etc.) will be provided to be prepared, cooked and shared by participants communally. The focus is on the recreational activity led by qualified coaches in a natural environment alongside veteran peers. At the end of the experience participants will be encouraged to create a ‘Facebook’ group in order to keep in contact via social media. At the end of the day, participants will be transported home by minibus.

A qualified mental health professional will be on site throughout to observe and monitor signs of distress, and if necessary to assist any participant who experiences flashbacks during the experience, but no formal psychological therapy will be offered or delivered during the intervention and there will be no deliberate initiation of discussions relating to trauma. The mental health professional will *respond to* questions about PTSD and provide some basic information and signposting to appropriate services if approached.

The angling context provides participants with tents and tackle situated around a lake. Participants are free to move around the lake and talk to other participants. The equine context involves participants collaborating in pairs to groom, ‘muck out’, prepare food and bedding and clean tack for their own horses. They are then taught riding skills before embarking on a horseback walk in surrounding fields. The falconry and archery context provides participants with a half day falconry and a half day archery in two groups, who swap activity at lunchtime. Participants learn how to handle and fly raptors and are coached in archery.

**Analyses**: In order to assess change in psychological wellbeing, two main approaches will be employed: 1) a mixed MANOVA with one between groups factor (intervention type: angling, equine, falconry) and repeated measures on all four psychological measures at each time point (3 time points: two weeks pre intervention, 2 weeks’ post intervention and 4 months post-intervention) will conducted on the data from all three interventions. 2) While statistical significance provides one index of change, it is also worthwhile to consider if the changes in PTSD symptoms observed might be considered clinically significant or reliable. For Clinically Significant Change (CSC) to be achieved, the level of functioning subsequent to the intervention should fall outside the range of the dysfunctional population, where range is seen as extending to two standard deviations beyond (in the direction of functionality) the mean of the population. The Reliable Change Index (RCI) is calculated using the change in a client’s score divided by the standard error of the difference for the measure(s) being used. The clinical significance of findings will be assessed both between the baseline and 2-week post intervention and the baseline and 4-month follow-up time points for all three groups.

**Participant Information & Consent Form**

**Description of the aim and procedure of the project**

The aim of this study is to look at the possible beneficial and therapeutic effects of outdoor experiences in a natural environment. To do this, you will be asked to fill in a small number of questionnaires to help us assess your current state of mind. Subsequently, you will be taken on either (1) an equine husbandry taster-day; (2) a falconry and archery day; or (3) a two-day angling trip involving an overnight stay. 14 days after the outdoor experience (and then subsequently 4 months), you will be contacted to fill in the questionnaires again.

**Confidentiality:**

Your part in this study is confidential. Your data are to be held confidentially and that only researcher and/or supervisor(s) will have access to them. Any personal information obtained in connection with this project and that can identify you will remain confidential. The data from the experiment will be held anonymously.

**Voluntary participation and withdrawal:**

Your participation in this experiment is voluntary; refusal to participate will involve no penalty. You may discontinue participation at any time or skip questions.

Before you make your decision, the researcher will be available so that you can ask any questions you have about the research project. You can ask for any information you want. Sign the Consent Form only after you have had a chance to ask your questions and have received satisfactory answers.

**Please read the statements below then sign and date the form if you consent to participate**

**I understand that:**

- My data are being collected as part of a University of Essex Research Project.
- My data are to be held confidentially and that only the researchers will have access to them.
- I have the right to withdraw my participation at any time and without giving any reason.
- I will be able to obtain a report of the results of this research.
- I am giving my consent for my data to be used for the following purposes: research
- Any questions I have about my participation have been answered.

***Signed: _____________________________________ Date: _________________***
