# Supplementary material for: Outdoor recreational activity experiences improve psychological wellbeing of military veterans with post-traumatic stress disorder: Positive findings from a pilot study and a randomised controlled trial
Source: PLoS One. 2020 Nov 25;15(11):e0241763. doi: 10.1371/journal.pone.0241763 (PMC7688151; doi:10.1371/journal.pone.0241763)
Supplement: S2 File — (DOCX) [file pone.0241763.s002.docx]

**Title: Exploring the effect of outdoor recreational activity experiences on the psychological wellbeing of military veterans with post-traumatic stress disorder (PTSD)**

**Names of researchers**: Dr Nicholas Cooper, Dr Mark Wheeler, Prof. Sheina Orbell, Dr Leanne Andrews, Prof. Jamie Hacker Hughes, Dr Tim Rakow, Dr Marie Juanchich.

**Background, Aims & Objections**: Exposure to the natural environment is being increasingly seen as a method for increasing wellbeing and psychological health in both clinical and non-clinical populations. Recently, reports in the literature also suggest that outdoor pursuits involving opportunities for social interaction and bonding may be particularly beneficial to Armed Forces Veterans experiencing Post-Traumatic Stress Disorder (PTSD). In particular, organized, social fishing trips have been shown to reduce psychological measures of stress in US veterans (Vella et al., 2013). Consequently, the current study aims to assess the impact of an outdoor pursuit programme (centred around social angling) on the psychological wellbeing of UK Veterans. Wellbeing is assessed in terms of PTSD symptoms, depression, anxiety, general social function, and stress.

**Hypotheses**: It is hypothesized that participants will experience a reduction in PTSD-related symptomology and an increase in subjective well-being as a consequence of a short, outdoor, recreational activity.

**Participants**: Inclusion criteria – military veterans with PTSD. Exclusion criteria – Not currently receiving psychological therapy for PTSD. Sample size – 25 (based on power calculation from pilot study. This indicated the a priori required sample size (alpha = 0.05 and power = .80) to detect an effect similar to that found in pilot study was 22, 11 participants per condition).

**Recruitment**: Participants to be recruited from a population of service users registered at local military welfare services.

**Design**: This is a waitlist controlled randomized experiment. It employs a pretest - posttest within participant design (time: pre-intervention, 2 weeks post-intervention, 4 months post-intervention) with one between groups factor (active intervention v waitlist control).

**Randomisation**: Participants will be randomly allocated to one of the two levels of the between groups factor (active intervention v waitlist control). They will be sequentially numbered and allocated to condition by means of an online blocked randomisation tool.

**Description of Procedure**: Dependent variables - The programme entails psychological profiling, in the form of the following questionnaire assessments: PCL-5 (post-traumatic stress disorder measure), GAD-7 (anxiety measure), PHQ-9 (depression measure), WSAS (work and social adjustment scale), PSS (perceived stress scale) at 2 weeks prior to intervention, 2 weeks and 4 months post intervention (see flow chart (Figure 1) for time points for both intervention and waitlist control groups).

The intervention protocol - Day 1 am – arrival at fishing lake, set-up equipment, health and safety briefing. Day 1 pm – fishing instruction, fishing & social interaction, evening meal. Day 2 am – fishing & social interaction. Day 2 pm – fishing & pack up equipment, instructions on how to keep in contact via social media etc.

The intervention details - The interventions are designed to deliver an outdoor recreational experience involving tuition in a peer group context. Attention is given to creating opportunities for participants to interact with each other. The venue will be made available exclusively to the veterans for the duration of the experience. Professional angling coaches provide instruction and are available at a ratio of two participants to one coach.

Participants will be transported to the venue by minibus or drive in their own car. On arrival at the venue, a health and safety briefing takes place. Participants are then allocated to coaches, provided with equipment (tents and tackle) and designated a fishing spot around a lake. Participants are free to move around the lake and talk to other participants. Participants collaborate in setting up a communal area for the purpose of socialising, eating and taking warm drink breaks. Food (e.g. sausages, burgers, chicken, salad etc.) is provided to be prepared, cooked and shared by participants communally. The focus is on the recreational activity led by qualified coaches in a natural environment alongside veteran peers. At the end of the experience participants will be encouraged to create a ‘Facebook’ group in order to keep in contact via social media. At the end of the day, participants are transported home by minibus or leave in their own vehicle.

A qualified mental health professional will be on site throughout to observe and monitor signs of distress, and if necessary to assist any participant who experienced flashbacks during the experience, but no formal psychological therapy was offered or delivered during the intervention and there was no deliberate initiation of discussions relating to trauma. The mental health professional may *respond to* questions about PTSD and provide some basic information and signposting to appropriate services if approached.

**Analyses**: Two main approaches: 1) The key analysis relates to the comparison of pre- (week 0) and post-intervention measures for the intervention and control groups taken at week 4 (see figure 1). A mixed MANOVA with one between groups factor (intervention vs. waitlist control) and repeated measures on all outcomes (baseline vs. 2 weeks post intervention) will be carried out. 2) While statistical significance provides one index of change, it is also worthwhile to consider if the changes in PTSD symptoms observed might be considered clinically significant or reliable. For Clinically Significant Change (CSC) to be achieved, the level of functioning subsequent to the intervention should fall outside the range of the dysfunctional population, where range is seen as extending to two standard deviations beyond (in the direction of functionality) the mean of the population. The Reliable Change Index (RCI) is calculated using the change in a client’s score divided by the standard error of the difference for the measure(s) being used. The clinical significance of findings will be assessed both between the baseline and 2-week post intervention and the baseline and 4-month follow-up time points.

**Figure 1**: Summary of Measurement and Intervention Timepoints for Intervention and Waitlist Control Group.

**Participant Information & Consent Form**

**Description of the aim and procedure of the project**

The aim of this study is to look at the possible beneficial and therapeutic effects of angling in a natural environment. To do this, you will be asked to fill in a small number of questionnaires to help us assess your current state of mind. Subsequently, you will be taken on a two-day angling trip involving an overnight stay. 14 days after the fishing trip (and then subsequently 4 months), you will again be contacted to fill in the questionnaires again.

**Confidentiality:**

Your part in this study is confidential. Your data are to be held confidentially and that only researcher and/or supervisor(s) will have access to them. Any personal information obtained in connection with this project and that can identify you will remain confidential. The data from the experiment will be held anonymously.

**Voluntary participation and withdrawal:**

Your participation in this experiment is voluntary; refusal to participate will involve no penalty. You may discontinue participation at any time or skip questions.

Before you make your decision, the researcher will be available so that you can ask any questions you have about the research project. You can ask for any information you want. Sign the Consent Form only after you have had a chance to ask your questions and have received satisfactory answers.

**Please read the statements below then sign and date the form if you consent to participate**

**I understand that:**

- My data are being collected as part of a University of Essex Research Project.
- My data are to be held confidentially and that only the researchers will have access to them.
- I have the right to withdraw my participation at any time and without giving any reason.
- I will be able to obtain a report of the results of this research.
- I am giving my consent for my data to be used for the following purposes: research
- Any questions I have about my participation have been answered.

***Signed: _____________________________________ Date: _________________***
