# Supplementary material for: Outdoor recreational activity experiences improve psychological wellbeing of military veterans with post-traumatic stress disorder: Positive findings from a pilot study and a randomised controlled trial
Source: PLoS One. 2020 Nov 25;15(11):e0241763. doi: 10.1371/journal.pone.0241763 (PMC7688151; doi:10.1371/journal.pone.0241763)
Supplement: Attachment — Submitted filename: response to reviewers letter.docx [file pone.0241763.s005.docx]

Dear Professor Moitra,

We thank the reviewers for their review of the manuscript and note that they are both content that the manuscript is technically sound, that the data support the conclusions, that the statistical analysis is appropriate and rigorous, that the data is fully available and that the manuscript is written in clear unambiguous English.

We also thank you for the opportunity to clarify the two remaining issues raised by Reviewer One. We provide our response below. We believe we have satisfactorily resolved the issues raised by Reviewer One. We have also taken this opportunity to complete a final review and proof read of the manuscript, since we are aware that the journal does not copy edit accepted manuscripts.

Reviewer #2: The authors have provided a comprehensive reply to the questions raised and also presented a greatly improved manuscript. The manuscript is well written, has important clinical message, and should be of great interest to the readers.

Author Response: We thank Reviewer Two for her positive comments about the manuscript including her observation concerning the importance of the clinical message and likely interest to your wider readership.

Reviewer #1: The authors have replied in detail and pertinently to all the reviewer’s points. There are just a couple of my points needing attention:

1. Original line 305 (now 346) Main effect of intervention group: the intervention effect is the interaction group x time, since we are looking for differences between the changes over time among the groups (compare statement of hypotheses in lines 213-215). The main effect includes baseline differences which are not a measure of intervention effect. Thus it should be clearly stated that the effect of the intervention(s) is measured and tested using the group x time interaction.

Author Response:

We believe that the reviewer has misunderstood or misread the design of experiment one. We believe that the analyses are correctly reported and interpreted in the current manuscript. The intervention effect is tested differently in experiment one and experiment two. To explain:

The design of experiment one is reported in the abstract (line 39: “experiment one employed a repeated measures design…”) and can also be viewed in Figure One. The statement of hypotheses relevant to experiment one appear on lines 209-211 and state that: “It was hypothesized that participants would experience a decrease in symptoms as assessed by established measures of PTSD, depression, anxiety and perceived stress as a result of the experience.” The reviewer 1 refers to the hypotheses of experiment two on lines 213-215. Experiment two was indeed a controlled experiment and the corresponding hypothesis was that experimental participants would experience a decrease in symptoms relative to controls. We have now made clear that the waitlist-controlled experiment is experiment two in the Aims by adding the words ‘experiment two’ on line 212.

In fact, in the previous round of reviews, Reviewer 2 (Methods: point one) had also sought clarification that in experiment one, all groups received an outdoor recreational intervention, that is, there was no control group as such, not an RCT. We confirmed that this is the case and that the between groups factor refers to the 3 different types of outdoor activity (lines 235-236). Consequently, from the mixed MANOVA, the important experimental effect that tests our hypothesis is the within-participant effect observed across all activity types as stated on line 347-348. Neither the statistical test of the between groups factor nor the group X time effect in this experiment test our hypothesis. The group X time interaction in this experiment (experiment one) tests whether the changes across time varied according to type of activity (because there is no control group as such). In other words, whether it made any statistical difference if participants experienced angling, or equine or falconry). It turned out that it did not (line 349-351).

Since the issue here seems to be merely one of stating clearly which is the important test of the hypothesis (as stated on line 343) we have sought to make this even clearer in the text:

“Participants’ mean psychological wellbeing before the intervention and at 2 weeks and 4 months after for each activity type are summarised in table 3. In order to assess the hypothesised change in psychological wellbeing from before to after the experience, a mixed MANOVA with one between groups factor (intervention type: angling, equine, falconry) and one within-participant factor (3 time points: two weeks pre-intervention, 2 weeks’ post intervention and 4 months post-intervention) was conducted on all four measures of psychological wellbeing. It was hypothesised that the analysis would reveal a significant within-participant effect.”

In experiment two, the reviewer is quite correct that the important intervention effect is the interaction of group x time because this analysis tests whether the change across time differed for the intervention group compared to the control group (see lines 496-7).

Reviewer 1:
2. Original line 316 Method for effect sizes. Lenhard and Lenhard is now cited, but the linked web-site provides several alternative definitions of effect size. I could not be sure which one was appropriate, and my attempts to verify this by calculating the effect sized did not yield values in agreement with those in Table 3. Which method was used?

Author Response:

Thank you for pointing out that readers may be unclear where to find this information on the cited website. We have now clarified.

The effect sizes shown in Table 3 are for pre-post pairwise comparisons of data. As the reviewer notes there are different methods for different types of data provided on <https://www.psychometrica.de/effektstaerke.html>

The effect size calculation we used was the one labelled 4: Effect sizes when measurement is repeated within a group (pre-post). We have added detail to the reference no. 56 (lines 816-8) so that together with the available raw data, a reader can calculate the effect sizes should they wish.

56.	Lenhard W, Lenhard A. Calculation of effect sizes: Dettelbach: Psychometrica; 2016 [Available from: <https://www.psychometrica.de/effektstaerke.html> Calculation of Effect Sizes 4. Effect sizes when measurement is repeated within a group (pre-post)]

Finally, we have also run all 4 figures through the PACE image correction software.

Kind regards, Nick Cooper (on behalf of the authors).
